# Diphtheria and Its Associates

**Published:** 1896-06

**Authors:** 


**Diphtheria and its Associates.** By LENNOX BROWNE. Pp. xii., 272. London: Baillière, Tindall, & Cox. 1895.

So many communications to the medical journals, both at home and abroad, have been made during the last few years on diphtheria that a book unencumbered with unnecessary details, written without bias, and yet containing the most recent theories and discoveries, would not be out of place. So vast, however, is the amount of material to draw from, that no one can hope to bring out a book of even moderate size and yet contain all that should be present. Mr. Browne has made a laudable attempt, and has produced a readable and, on the whole, fairly complete treatise on diphtheria and its associates. His object has been throughout, as he tells us in his prefatory remarks, to indicate the necessity for the scientific expert and the clinician to work together, an essential concerning which all are now agreed. But we are inclined to think that the clinician is more frequently a general practitioner than a specialist on the throat; and in this opinion we are strengthened by Mr. Browne's admission that but for the kindly assistance of the Metropolitan Asylums' Board, his clinical experience of the disease would be small.

In such a book there must be many points on which different opinions might be held, and in a careful perusal we have come across several. We do not particularly refer by this to the treatment by antitoxin, which Mr. Browne relegates to the appendix, for since the publication of the book much additional evidence of its value has been brought out, but to many other little matters of detail of minor importance. For instance, Mr. Browne clearly distinguishes between diphtheria and pseudo-diphtheria, but appears to ignore the occurrence of a bacillus sufficiently resembling the true one in appearance to cause a little difficulty in diagnosis. He assumes that pseudo-diphtheria is a condition brought about by a micrococcus quite unmistakable with Löffler's bacillus; but the occasional occurrence of a bacillus which, though it may have nothing to do with the morbid condition, so far resembles Löffler's as to have earned the name of pseudo-diphtheriticus is certainly worthy of some notice.

The illustrations are excellent, but would have been of more value to those inexperienced in bacteriology if the magnifying power had been stated.

